# Assessing the quality of life among victims of spinal occupational injuries

**DOI:** 10.1192/j.eurpsy.2025.1632

**Published:** 2025-08-26

**Authors:** A. Hrairi, N. Rmadi, A. Kchaou, A. Haddar, M. Hajjaji, K. Jmal Hammami

**Affiliations:** 1 Occupational medicine department, Hedi chaker university hospital, University of Sfax, Sfax, Tunisia

## Abstract

**Introduction:**

Spinal occupational injuries constitute an important health problem affecting workers in their most productive years. The outcomes of these injuries may influence the quality of life of those victims.

**Objectives:**

Assessing the quality of life among victims of spinal occupational injuries and identifiying the associated factors to physical and mental health.

**Methods:**

A cross-sectional analysis was conducted during 9 months (from October 2020) among workers victims of occupational injuries consulting for an Impairment Rating Evaluation. Only those with spinal injuries were included. Socio-professional data and the accident’ outcomes were collected. The assessment of perceived mental health (Mental Component Summary: MCS) and physical health (Physical Component Summary: PCM) by each employee was obtained by the 12-item Short Form Health Survey. Social support was evaluated by the Social Support Scale. The pain was evaluated by a Visual Analogue Scale. Unsuccessful return to work comprises all situations other than a satisfactory return to the same position held before the accident.

**Results:**

A total of 73 injured workers were included, 75.3% were male. The mean age was 42.78± 8.46 years. The mean job seniority was 14.44±7.86 years and the average number of working hours per week was 46.93±5.61 hours. Among the participants, 43.8% had an unsuccessful return to work, 28.1% of them had a low social support. The mean scores for MCS and PCS were 41.17± 11.89 and 29.57± 5.31 respectively.

Factors assocoiated with MCS were : the duration of absence (p=0.008), the rate of Permanent Partial Disability (p=0.025), lhe low social support (p=0.000), working in confection sector (p=0.016) and unsuccessful return to work (p=0.023).

Factors associated with PCS were : The pain score (p=0.002), the duration of immobilisation (p=0.007) and being a laborer (p=0.002). No association was found with age, job seniority, the presence of disc diseases and hospitalisation duration.

**Conclusions:**

Victims of spinal occupational injuries are vulnerable to unfavorable outcomes from work accidents, and hence the need for special efforts to reintegrate them into professional life.

**Disclosure of Interest:**

None Declared

